# Glucagon-Like Peptide-1 (GLP-1) Receptor Agonists in Diabetes and Obesity: Implications for Periodontology and Family Dentistry

**DOI:** 10.7759/cureus.95792

**Published:** 2025-10-31

**Authors:** Asmaa A Almohammad, Sara K Aldossari, Ziyad A Sukumbaji, Raniah A Faden, Mona A Almutairi, Noura H Al-Harbi, Majida Y Al-Saaib, Bandary S Almarshedy, Entesar A Alharabi, Wafaa A Alhazri, Abdulaziz M Altalhi

**Affiliations:** 1 Faculty of Dentistry, Alneelain University, Khartoum, SDN; 2 Department of General Dentistry, Kharsa Clinics, Riyadh, SAU; 3 Department of General Dentistry, Ministry of Health, Makkah, SAU; 4 Department of General Dentistry, Ministry of Health, Riyadh 2nd Cluster, Riyadh, SAU; 5 Division of Periodontology, Department of Dentistry, Ministry of Health, Riyadh 2nd Cluster, Riyadh, SAU; 6 Division of Family Dental Medicine, Department of Dentistry, Ministry of Health, Riyadh 2nd Cluster, Riyadh, SAU

**Keywords:** diabetes mellitus, glp-1 receptor agonists, inflammation, obesity, periodontal disease

## Abstract

Periodontitis, an immune-inflammatory disease triggered by a dysbiotic microbial complex, often leads to the destruction of periodontal supporting tissues. It has a bidirectional association with systemic comorbidities, such as type 2 diabetes mellitus (T2DM) and obesity, through chronic low-grade inflammation. Glucagon-like peptide-1 (GLP-1) is an incretin hormone that regulates glucose metabolism, lipid homeostasis, and other vital biological functions. By promoting insulin secretion from pancreatic β-cells, GLP-1 maintains glucose levels, body weight, appetite, and immune function. Accordingly, GLP-1 receptor agonists (GLP-1RAs) are widely used in the treatment of T2DM and have recently been adopted for obesity management. Furthermore, GLP-1RAs exhibit anti-inflammatory, antioxidant, osteoprotective, and anti-matrix metalloproteinase properties, which may help protect against and slow the progression of periodontitis. These effects are particularly valuable in patients with coexisting diabetes and obesity, providing synergistic benefits for systemic metabolic regulation and periodontal health. However, there is a lack of direct randomized clinical trials evaluating periodontal endpoints, such as bleeding on probing, probing depth, clinical attachment level, and radiographic bone changes. Therefore, any periodontal benefits attributed to GLP-1RAs should be regarded as preliminary evidence, supported mainly by preclinical studies and indirect metabolic effects.

## Introduction and background

Periodontitis is a chronic infectious-inflammatory disease characterized by periodontal attachment loss and alveolar bone destruction. It results from immune-inflammatory responses triggered by host-periodontopathic bacterial interactions [1]. Marked by low-grade systemic inflammation, periodontitis involves the persistent subclinical release of inflammatory mediators into the circulation [2]. If left untreated, this low-intensity inflammation can escalate into a significant systemic inflammatory state, which can exacerbate the bidirectional relationship between periodontitis and systemic conditions such as obesity [3] and insulin resistance [4].

The inflammatory mediators interleukin-1 beta (IL-1β), interleukin-6 (IL-6), and tumor necrosis factor-alpha (TNF-α) [5,6] stimulate immune functions at both the gingival and systemic levels [7]. Additionally, the concentrations of C-reactive protein (CRP) and prostaglandin E2 (PGE2) increase in accordance with the release of proinflammatory cytokines. These inflammatory markers are detected in both periodontitis and systemic conditions such as diabetes, obesity, and arthritis [8]. Clinically, this helps explain why obese and diabetic patients often demonstrate more severe periodontal breakdown and impaired healing responses compared to healthy individuals [7-13].

Insulin resistance (IR), caused by persistent low-intensity inflammation, disrupts cytokine regulation within periodontal tissues [9,10]. This contributes to impaired β-cell function, IR, endothelial dysfunction, and progression toward diabetes and atherosclerosis [7]. In addition, IR is a hallmark of obesity and may serve as a potential predictor of gingival and periodontal inflammation in obese individuals [11]. In obese patients, adipose cells and macrophages within adipose tissue secrete TNF-α and IL-6; these adipocytokines play a pivotal role in the link between obesity and periodontitis [12,13]. Adipokines impair insulin sensitivity and glucose homeostasis via chemokine signaling pathways, thereby modulating the systemic inflammatory state. IL-6 promotes fatty acid oxidation and CRP production by the liver while suppressing insulin signaling. TNF-α facilitates lipolysis and increases IL-6 expression in adipose cells; together, these contribute to IR [14,15]. Thus, periodontitis, diabetes, and obesity form a complex, bidirectional triad, with each element influencing the onset and progression of the others.

The incretin hormone GLP-1 is produced through the enzymatic cleavage of proglucagon and is secreted by intestinal L-cells in response to nutrient intake. It plays a critical role in blood glucose regulation by stimulating insulin synthesis from pancreatic β-cells [16,17]. The GLP-1 receptor (GLP-1R), a member of the G-protein-coupled receptor (GPCR) family, is located on the membranes of various cell types. GLP-1R is expressed not only in the pancreas but also in multiple organs throughout the body [18,19], and it regulates appetite via the central nervous system. Deficiency of GLP-1R signaling, therefore, contributes to weight gain and obesity by increasing appetite [20]. Moreover, GLP-1R maintains glucose homeostasis by balancing insulin and glucagon secretion [21,22]. Given these diverse roles, GLP-1 signaling occupies a central position at the intersection of metabolism, immunity, and tissue repair - all of which are directly relevant to periodontal health [7,12,21].

GLP-1RAs are synthetic protein molecules that mimic the physiological functions of GLP-1 by having amino acid sequences similar to native GLP-1 [23]. In addition to improving glucose homeostasis by stimulating insulin release and suppressing glucagon secretion, GLP-1RAs delay gastric emptying, prolong satiety, reduce appetite, and promote weight loss [24]. For example, the GLP-1RA liraglutide is widely used for the therapeutic management of T2DM and obesity [25,26]. Despite these promising systemic effects, the direct periodontal impact of GLP-1RAs remains underexplored, and current evidence is largely preclinical or observational [25,26]. This underscores the need for focused clinical trials assessing periodontal outcomes in patients receiving GLP-1RAs.

This review aims to elucidate the pathophysiological role of GLP-1RAs in the interconnected triad of periodontitis, diabetes, and obesity, with emphasis on their roles in maintaining periodontal health. However, much of the supporting evidence is preclinical or inferential. Any putative periodontal benefits of GLP-1RAs should therefore be interpreted cautiously and further validated through randomized human trials directly assessing periodontal endpoints, such as bleeding on probing, probing depth, clinical attachment level, and radiographic bone changes.

## Review

Research methodology

A comprehensive literature search was conducted using major electronic databases, including PubMed, Scopus, Google Scholar, and ScienceDirect. The keywords used were “GLP-1 receptor agonists,” “GLP-1RA,” “periodontitis,” “GLP-1RA and diabetes,” “GLP-1RA and obesity,” “GLP-1RA and oxidative stress,” “GLP-1RA and periodontitis,” “GLP-1RA and inflammation,” and “GLP-1RA and mechanism of action.”

Relevant articles were selected after careful review of titles and abstracts. Peer-reviewed research papers, review articles, and both in vivo and in vitro studies published in English were included. While the focus was on studies linking GLP-1RAs with periodontal health, we also considered studies that addressed their broader roles in diabetes, obesity, and related inflammatory or bone pathways. Studies unrelated to GLP-1RAs or the periodontitis-diabetes-obesity triad, as well as non-English or abstract-only papers, were excluded.

As this is a narrative review, the focus was on synthesizing recent and relevant studies up to 2025 rather than applying systematic protocols. Priority was given to high-quality and clinically meaningful evidence, with redundancy minimized to maintain clarity and highlight future research directions.

Mechanism of GLP‑1 and GLP‑1RAs: Signal transduction and systemic effects

Signal Transduction Basics

GLP-1Rs are Class B G-protein-coupled receptors primarily coupled to Gs. Their activation increases intracellular cAMP and engages protein kinase A (PKA) and EPAC (Exchange Protein Directly Activated by cAMP). Cross-talk with phosphoinositide-3-kinase/protein kinase B pathway (PI3K/Akt) and AMP-activated protein kinase (AMPK) pathways converges on reduced nuclear factor kappa-B (NF-κB) activity and oxidative stress, which are mechanisms implicated in periodontal immune modulation, matrix turnover, and bone remodeling [27-40].

Pancreatic β-Cell Effects (Insulinotropic and Anti-apoptotic Pathways)

GLP-1 initiates its signaling cascade by binding to GLP-1R, a Class B GPCR [27,28]. Following G-protein stimulation, intracellular cAMP (cyclic adenosine monophosphate) levels increase [28-30], and this in turn activates protein kinase A (PKA). PKA enhances insulin biosynthesis and secretion while inhibiting glucagon release [31,32]. Rap1, activated by cAMP through the EPAC pathway [33-36], further regulates insulin secretion.

GLP-1 supports the survival and function of pancreatic β-cells by activating the phosphoinositide 3-kinase (PI3K)/protein kinase B (Akt) pathway [37-40]. Together, the cAMP-PKA/EPAC and PI3K/Akt branches provide acute insulinotropic effects alongside trophic support for β-cell health [37-41]. GLP-1 also enhances insulin signal transduction and improves peripheral tissue sensitivity to insulin via the PI3K/Akt pathway [20,41]. In addition, it reduces hepatic glucose synthesis by inhibiting key gluconeogenic enzymes [42-44].

Once activated, Akt enhances cellular glucose uptake by increasing the expression and translocation of Glucose Transporter Type 4 (GLUT-4) to the cell membrane [45-47]. Akt also promotes cell survival by phosphorylating and inhibiting pro-apoptotic proteins such as Bad and members of the FOXO family [47-49]. Through these mechanisms, GLP-1 prevents apoptosis and preserves β-cell mass and function over time [37-40,47-49].

Regulation of Glucagon and Somatostatin

Glucagon, synthesized by pancreatic α-cells, is suppressed by GLP-1 via multiple regulatory mechanisms. For instance, GLP-1 inhibits glucagon secretion by directly binding to receptors on α-cells [50,51]. Since glucagon promotes hepatic glucose release, suppressing its secretion is critical for lowering blood glucose levels. GLP-1 also stimulates insulin secretion from β-cells [52,53], thereby reducing blood glucose and further suppressing glucagon release via a feedback mechanism [54]. Together, these actions establish an accelerator-brake system that tightly regulates glycemia [50,51,54].

Additionally, GLP-1 prolongs gastric emptying and reduces appetite, further reducing glucose levels [55,56]. Somatostatin, secreted primarily by δ-cells following GLP-1R activation, directly inhibits glucagon secretion from adjacent α-cells [57]. It also indirectly suppresses pancreatic hormone secretion, gastric acid production, and gastrointestinal activity, thereby reducing glucagon demand [58,59]. While α-cells express low levels of GLP-1R [60], δ-cells express higher levels [61], making somatostatin-mediated suppression the predominant mechanism by which GLP-1 inhibits glucagon secretion. In T2DM, GLP-1RAs utilize this δ-cell-mediated pathway to exert their effects [57-61].

CNS Effects

GLP-1 reduces appetite, and therefore food consumption, by acting on the central nervous system [62]. Within the hypothalamus, GLP-1R activation stimulates satiety-promoting pro-opiomelanocortin/cocaine- and amphetamine-regulated transcript (POMC/CART) neurons and inhibits hunger-promoting neuropeptide Y/agouti-related peptide (NPY/AgRP) neurons [63,64]. GLP-1 also acts on receptors in the gut to prolong gastric emptying by reducing gastric smooth muscle contractions, thereby extending gastric retention time [65,66]. Additionally, GLP-1 reduces gastric contractions through vagal nerve activation, contributing to delayed gastric emptying [66,67]. This prolongation of gastric retention enhances satiety and reduces caloric intake [68,69].

GLP-1 also supports long-term weight management by regulating postprandial glucose levels through delayed gastric emptying [27]. As these systemic effects of lower HbA1c and weight loss can indirectly influence periodontal outcomes, it is important to take these factors into account in clinical situations [55-56,66-69].

Indirect Metabolic Pathways

GLP-1RAs may indirectly reduce periodontal risk through several mechanisms: improved glycemic control (via PI3K/Akt insulin signaling), reduced adipose-derived cytokine release, attenuation of systemic inflammatory tone, and mitigation of AGE-RAGE-mediated oxidative stress. These systemic effects may lower gingival inflammatory burden and enhance the patient’s response to conventional periodontal therapy [20,41,55-56,66-69].

Table 1 outlines the principal systemic and molecular pathways modulated by GLP-1RAs and their inferred impact on periodontal tissues, highlighting expected clinical endpoints, levels of supporting evidence, and key confounders.

**Table 1:** Mechanistic pathways of GLP-1/GLP-1 receptor agonists and their systemic/periodontal effects. Arrows denote direction of effect (↑ increase; ↓ decrease). BOP, bleeding on probing; PD, probing depth; CAL, clinical attachment level; NF-κB, nuclear factor kappa-B; ROS, reactive oxygen species; NO, nitric oxide; AGE-RAGE, advanced glycation end-products/receptor for AGE axis; ADMA, asymmetric dimethylarginine; cAMP, cyclic adenosine monophosphate; PKA, protein kinase A; EPAC, exchange protein directly activated by cAMP; Rap1, Ras-related protein 1; PI3K/Akt, phosphoinositide-3-kinase/protein kinase B pathway; GLUT-4, glucose transporter type 4; Bad/FOXO, pro-apoptotic Bcl-2-associated death promoter/Forkhead box O transcription factors; α/δ-cells, pancreatic alpha/delta islet cells; HbA1c, glycated hemoglobin; ACEi, angiotensin-converting enzyme inhibitor. Evidence tier: Preclinical (in vitro/animal); Human metabolic (inferred perio) = human data showing metabolic/biomarker changes with periodontal effects inferred rather than directly measured; Observational human (inferred perio) = human cohort/case-control without randomized periodontal endpoints; Biomarker human = human studies reporting periodontal or systemic biomarkers. *Expected periodontal endpoints* indicate hypothesized clinical changes based on the cited mechanisms; definitive periodontal randomized controlled trial (RCTs) are pending. *Confounders to adjust* lists variables recommended for adjustment in analyses/interpretation (e.g., HbA1c, weight, smoking, concomitant medications).

Pathway/node	Systemic metabolic effect (↑/↓)	Periodontal target cells/processes	Expected periodontal endpoints	Evidence tier	Confounders to adjust
cAMP-PKA/EPAC signaling [27-36]	↑ Insulin biosynthesis and secretion; ↓ glucagon; EPAC→Rap1 exocytosis	Indirect: lowers hyperglycemia-driven inflammatory stress (↓ NF-κB/ROS load)	BOP ↓, PD ↓, CAL ↑ (indirect via glycemia)	Preclinical; Human metabolic (inferred perio)	HbA1c, weight, concomitant meds (statins/ACEi)
PI3K/Akt (incl. anti-apoptotic Akt) [20,37-41,47-49]	↑ β-cell survival; ↑ insulin sensitivity; ↓ hepatic glucose output; ↑ GLUT-4; ↓ Bad/FOXO	Indirect: ↓ AGE-RAGE oxidative stress; supports bone remodeling milieu	BOP ↓, PD ↓, CAL ↑; bone preservation (indirect)	Preclinical; Human metabolic (inferred perio)	HbA1c, weight
Islet hormone modulation (α/δ): glucagon suppression + δ-cell somatostatin [50-54,57-61]	↓ Glucagon directly and via ↑ somatostatin; smoother post-prandial excursions	Indirect: ↓ AGE formation → ↓ AGE-RAGE signaling in periodontal tissues	BOP ↓, PD ↓ (indirect via glycemic stability)	Preclinical; Human metabolic (inferred perio)	Post-prandial glycemia, weight
Central/vagal effects & gastric emptying [62-69]	↑ Satiety; ↓ intake; ↓ gastric emptying → ↓ post-prandial spikes	Obesity-related risk ↓; lower glycemic variability burden on the gingiva	BOP ↓, PD ↓ (indirect via weight & glycemia)	Preclinical; human metabolic	Weight change (must adjust)
Indirect systemic effects (glycemia, cytokines, oxidative stress) [20,41,55-56,66-69]	↓ Adipose cytokines; ↓ systemic inflammatory tone	↓ Gingival inflammatory burden; better response to periodontal therapy	BOP ↓, PD ↓, CAL ↑	Preclinical; Observational human (inferred perio)	HbA1c, weight, smoking
AGE-RAGE/ADMA/ROS axis (antioxidant) [70-94]	↓ ADMA; ↑ NO bioavailability; ↓ ROS and aberrant autophagy	↓ Collagenolysis/oxidative damage; supports soft-tissue/bone preservation	BOP ↓, PD ↓; bone loss ↓	Preclinical; Biomarker human (ADMA)	HbA1c, lipids, antihypertensives

Anti-inflammatory, antioxidant, anti-matrix metalloproteinases (MMP), and bone-protective effects of GLP-1RAs

Direct Periodontal Tissue Actions (Local Cell/Tissue Effects)

Preclinical models and cell studies indicate that GLP-1R signaling favors M2 macrophage polarization, reduces NF-κB-driven cytokines (IL-1β, IL-6, TNF-α) and ROS, and lowers the receptor activator of NF-κB ligand/osteoprotegerin (RANKL/OPG) ratio, thereby suppressing osteoclastogenesis. In periodontal ligament and osteoblast-lineage cells, GLP-1R signaling intersects with Wnt/β-catenin and MAPK pathways to support osteogenic differentiation and matrix maintenance [70-80].

Overview and Periodontal Relevance

GLP-1RAs exhibit anti-inflammatory [70], osteoprotective [71-73], antioxidant, and anti-MMP properties, in addition to their well-established roles in glucose homeostasis, delayed gastric emptying, promotion of satiety, and long-term obesity management. These properties are directly relevant to periodontal tissue preservation, as inflammation, oxidative stress, and matrix degradation can lead to attachment loss and bone resorption [78,79,89,90].

Modulation of Inflammatory Pathways

GLP-1Rs are evident in immune cells, such as macrophages, monocytes, and lymphocytes. Therefore, when ligating with this receptor, GLP-1RAs exert an anti-inflammatory response by controlling signaling within immune cells [74]. These immune cell populations are abundant in periodontal lesions and provide a plausible local target for GLP-1RAs [78,79].

NF-κB, or nuclear factor-kappa-light-chain-enhancer of activated B cells, is a key transcription factor that regulates the inflammatory process [75]. GLP-1RAs inhibit NF-κB activation, thereby suppressing the secretion of proinflammatory cytokines and adhesion molecules that mediate immune cell attraction to inflammation sites [76]. Furthermore, GLP-1RAs weaken the NF-κB pathway signaling, thus reducing the severity of the inflammatory response [74]. GLP-1RAs also restricts the secretion of key inflammatory mediators TNF-α, IL-6, and IL-1β [77]. The major activators of NF-κB, such as bacterial lipopolysaccharide (LPS), PGE2, IL-1β, TNF-α, stress, and viral agents, are highly evident in periodontal disease [78]. In a study, NF-κB activation was significantly upregulated in patients with chronic periodontitis compared to healthy controls [79]. Therefore, NF-κB plays a critical role in the pathoetiology of periodontal disorders, and its suppression by GLP-1RAs would be expected to reduce BOP and pocket inflammation in clinical settings [78,79].

In addition to inhibiting NF-κB, GLP-1RAs activate AMPK, which exerts anti-inflammatory effects [74]. The AMPK pathway is central to cell metabolism and the inflammatory response [80]. Its activation attenuates proinflammatory signaling and suppresses mediator and chemokine secretion [74]. AMPK thus provides a mechanistic bridge linking metabolic control to inflammation resolution in periodontal tissues [80-82]. Stimulating AMPK activity mitigates M1 macrophage polarization and reduces the secretion of proinflammatory cytokines (TNF-α, IL-6, and IL-1β) [81]. Conversely, experimental periodontitis was found to worsen in AMPK knockout mice [82]. GLP-1RAs also upregulate the anti-inflammatory cytokine IL-10, enhancing their immunomodulatory potential [83]. This anti-inflammatory shift is consistent with reduced osteoclastogenic signaling via a favorable RANKL/OPG balance [85,86].

The GLP-1RA liraglutide attenuates inflammatory gene expression, inflammatory cell infiltration in the gingiva, and alveolar bone loss in ligature-induced periodontitis. It reduces M1 macrophages in gingiva and alveolar bone by downregulating osteoclast development, thereby slowing periodontitis progression. However, liraglutide does not upregulate osteoblast activity [84]. Despite this, the net effect favors bone preservation through reduced resorption, leading to reduced alveolar bone loss [84]. Since TNF-α stimulates osteoclastogenesis directly or indirectly, GLP-1-mediated suppression of TNF-α contributes to osteoclast downregulation [85,86], reinforcing the link between gingival inflammation and alveolar bone resorption. Clinically, this pattern would translate into reduced probing depths (PDs) and stabilized attachment levels [84-86].

In vitro studies on human PDLSCs exposed to LPS showed that liraglutide promotes migration, proliferation, and osteogenic differentiation while reducing TNF-α and IL-6 secretion. Mineralization nodules were also formed via upregulated bone markers (ALP, Runx2) [87]. Porphyromonas gingivalis LPS suppressed bone formation and activated Wnt/β-catenin signaling, but these effects were counteracted by liraglutide. This reduced alveolar bone loss, decreased inflammatory cytokines (TNF-α, IL-1β, IL-6), and limited the infiltration of inflammatory cells [88]. Restoration of osteogenic markers alongside reversal of Wnt/β-catenin perturbation, therefore, supports a regenerative-leaning environment and predicts improvements in CAL and alveolar bone stability [87,88].

Exenatide downregulates IL-1β, inducible nitric oxide synthase (iNOS), and MMP-9 transcription in gingival tissues with periodontitis [89]. Furthermore, MMP-9 reduction may limit collagen breakdown and connective tissue attachment loss [89]

Antioxidant Effects and Oxidative Stress

GLP-1RAs mitigate oxidative damage by reducing reactive oxygen species (ROS) generation, thereby preventing chronic inflammation and tissue injury [90]. Given the high ROS burden in periodontal lesions, this mechanism supports the preservation of soft tissue and bone [90]. In diabetes, GLP-1RAs suppress ROS production by inhibiting asymmetric dimethylarginine (ADMA) via the AGE-RAGE signaling axis and by downregulating protein arginine methyltransferase-1 [91]. They also inhibit ROS-induced abnormal autophagy and inflammation through histone deacetylase 6 (HDAC6) activity, which is mediated by the GLP-1R-ERK1/2 pathway [92].

These oxidative stress pathways are strongly implicated in periodontal pathology, suggesting that GLP-1RA-mediated ROS control has clinically evident benefits [90-92]. ADMA, an endogenous nitric oxide synthase (NOS) inhibitor, is elevated in both diabetes and chronic periodontitis. It contributes to endothelial dysfunction, oxidative stress, and impaired nitric oxide activity. It is also considered a potential biomarker for periodontitis, cardiovascular disease, and diabetes [91,93,94]. ADMA levels in gingival crevicular fluid (GCF) are significantly higher in individuals with periodontitis and positively correlate with IL-1β and clinical periodontal parameters [94]. Therefore, GLP-1RAs may provide dual therapeutic benefits in reducing oxidative stress in both diabetes and periodontitis. Subsequently, lowering ADMA/AGE-RAGE signaling is expected to reduce bleeding on probing (BOP), PD, and alveolar bone loss [90-92,94].

Influence on Bone Metabolism: Osteoblast and Osteoclast Pathways

Normal bone turnover requires a balance of formation and resorption, and it involves osteoblasts, osteoclasts, and osteocytes within the bone matrix [95]. Mesenchymal stem cells (MSCs) can differentiate into either osteoblasts or adipocytes [96,97], but liraglutide biases MSC differentiation toward osteoblasts. This enhances bone formation [98], and it may support regeneration in periodontal defects and reduce alveolar bone loss [98].

The mitogen-activated protein kinase (MAPK) and protein kinase C (PKC) pathways regulate MSC differentiation into adipocytes, but GLP-1 stimulates MSC proliferation and restricts premature adipogenesis [99]. Instead, GLP-1 directs lineage commitment toward osteoblasts by modulating MAPK and Wnt signaling, thereby upregulating Runx2, a master transcription factor for osteoblast differentiation [100,101]. Additional pathways implicated include PKA/β-catenin and PKA/PI3K/AKT/GSK-3β [102]. Runx2 upregulation is associated with changes relevant to clinical attachment gain, such as increased ALP, collagen type I, and mineralization [101,102].

Wnt signaling is also central to GLP-1-mediated osteogenesis. The canonical Wnt pathway (low-density lipoprotein receptor-related protein 5/6 (LRP5/6), β-catenin, glycogen synthase kinase-3 beta (GSK-3β), and T-cell factor (TCF)) promotes osteoblast differentiation and maturation [103,104,105]. GLP-1 functions as an upstream activator of this pathway [103-106]. In diabetic rats with impaired Wnt activity, exendin-4 was found to enhance bone formation [103]. Accordingly, restoration of Wnt signaling in diabetic periodontitis may help preserve crestal bone height [103-106].

The SOST gene encodes sclerostin, an osteocyte-secreted glycoprotein that suppresses bone formation by inhibiting BMP activity, ALP expression, collagen synthesis, and mineralization [107]. By attenuating Wnt/β-catenin signaling, sclerostin acts as a negative regulator of bone metabolism [108]. Exendin-4 has been shown to suppress sclerostin expression, thereby enhancing bone formation via Wnt/β-catenin signaling in osteocytes [109]. Thus, sclerostin suppression potentially allows GLP-1RAs to improve alveolar bone density in periodontitis [107-109].

GLP-1 also synergizes with adenosine triphosphate (ATP) to support c-Fos transcriptional activity in osteoblasts and enhance osteogenesis [110]. Liraglutide promotes osteogenesis in MC3T3-E1 osteoblasts through ERK1/2, PI3K/AKT, and cAMP/PKA/β-catenin (Ser675) signaling [73]. Activation of these ERK/AKT/β-catenin nodes aligns with periodontal ligament stem cell (PDLSC) osteogenesis and the Runx2-ALP axis described above [73,87,100].

Osteoclast differentiation depends on TNF-α family mediators, such as RANK, RANKL, and OPG. These regulate precursor differentiation, activation, and apoptosis, with RANKL expression on osteoblasts driving osteoclastogenesis [111,112]. In ovariectomized rats, exendin-4 increases OPG mRNA and suppresses RANKL mRNA expression, thus reducing bone resorption [72]. Accordingly, a lower RANKL/OPG ratio predicts fewer TRAP-positive osteoclasts and reduced alveolar bone resorption [72,84].

GLP-1 also improves calcitonin release from thyroid C cells via cAMP signaling [113,114]. In animal models, exendin-4 upregulated calcitonin expression in wild-type mice but not in GLP-1R-deficient mice, which instead showed increased urinary deoxypyridinoline (DPD). Furthermore, calcitonin supplementation in GLP-1R-deficient mice reduced DPD levels, confirming a calcitonin-dependent antiresorptive mechanism [115]. Similarly, exendin-4 attenuated LPS-induced bone resorption by reducing osteoclast numbers, C-terminal telopeptides (CTX), and resorption pits [116]. This calcitonin-mediated pathway likely complements local periodontal effects, particularly in inflammatory conditions [115,116].

Exendin-4 also promotes PDLSC proliferation and osteogenic differentiation while mitigating glucose-induced cytotoxic effects [117,118]. It enhances bone strength, prevents trabecular bone loss, and promotes bone formation in ovariectomized rats [72]. Further studies have shown that GLP-1RAs reverse glucose-induced suppression of PDLSC osteogenesis via MAPK and Wnt signaling [119]; this supports its therapeutic use in both T2DM and periodontitis [119,120]. Collectively, these findings suggest GLP-1RAs could serve as adjunctive agents to enhance regeneration in periodontitis patients with metabolic comorbidities [117-120].

Finally, liraglutide administration in diabetic rats with ligature-induced periodontitis reduced bone loss, improved microarchitecture, and attenuated periodontal inflammation. It not only lowered blood glucose but also reduced the RANKL/OPG ratio and downregulated IL-6, TNF-α, and IL-1β [121]. These dual metabolic and bone-immune effects suggest clinical potential to improve attachment level and crestal bone height, although confirmation in human trials is required [121].

Anti-MMP Activities of GLP-1RAs

MMPs are enzymes that degrade the components of the extracellular matrix. GLP-1RAs such as exenatide, liraglutide, and semaglutide downregulate MMP expression [122-125]. In periodontal tissues, this downregulation is expected to attenuate collagen degradation and preserve connective tissue attachment [126-133].

In atherosclerosis, excess MMP activity contributes to fibrous cap thinning and plaque rupture [122]. Similarly, in the periodontium, excessive MMP activity leads to connective tissue breakdown and pocket deepening [127-135]. GLP-1 administration suppresses MMP-3 and MMP-13, which are upregulated in osteoblasts by IL-1β [125]. Dampening MMP-3/13 may limit inflammatory matrix turnover adjacent to alveolar bone [125,130,131].

In patients with type 1 diabetes, both acute hyperglycemia and hypoglycemia can cause surges in MMP-9, likely driven by oxidative stress. GLP-1 counteracts these effects, presumably through its antioxidant properties [126]. By constraining oxidative-stress-driven MMP-9 surges, GLP-1 may blunt episodic collagenolysis during periodontal exacerbations [126,127,132].

Several MMP classes are expressed in gingival tissues. In GCF, the most common are MMP-8, MMP-9, and MMP-13 [127-130]. Among collagenases, MMP-8 accounts for nearly 80% of total collagenase activity in GCF, while MMP-13 and MMP-1 contribute smaller amounts in chronic periodontitis [131]. MMP-8, a neutrophil collagenase, is responsible for degradation of gingival and periodontal ligament collagen [132,133]. Because of this, GCF MMP activity serves as a noninvasive biomarker for monitoring host-modulating therapies such as GLP-1RAs [134,135].

In patients with moderate to advanced periodontitis, GCF MMP-9 and MMP-13 levels have been shown to be significant predictors of disease progression over a two-month monitoring period [134,135]. Furthermore, MMP-9, which is abundantly expressed in osteoclasts, plays a key role in bone resorption [136,137]. Accordingly, GLP-1RA-associated reductions in MMP-9 can decrease osteoclastic activity and bone loss [122-124,136,137].

Although some studies report contradictory findings regarding the precise role of MMP-9 in bone resorption [138], most studies support its importance [139]. Regardless of this debate, reducing MMP-9 and MMP-8 activity is directionally favorable for periodontal stability [127-135].

Further studies specifically evaluating the effects of GLP-1RAs on MMP activity in periodontitis are needed. Moreover, future clinical trials should prespecify GCF MMP-8/9/13, along with BOP , PD , clinical attachment level (CAL), and radiographic bone outcomes, as co-primary endpoints [134,135].

Biological Pathways and Clinical Outcomes

The convergence of immunomodulatory pathways (NF-κB suppression and M1-to-M2 polarization), antioxidant mechanisms (reduction of ROS and ADMA), and osteoregulatory signaling (RANKL/OPG balance, Wnt/β-catenin activation, and ERK/AKT signaling) establishes a strong biological rationale for enhanced periodontal stability. Collectively, these mechanisms support potential reductions in BOP, PD, and CAL, as well as preservation of crestal bone. However, confirmation of this potential requires well-designed human trials [72-79,81,85-88,90-92,100,103,116,121].

GLP-1RAs therefore present a dual therapeutic pathway, comprising systemic metabolic improvement in combination with local immune and bone modulation, that may translate into improved periodontal outcomes. However, definitive randomized human studies with periodontal endpoints remain essential [72,84,103,115,116,121]. Table 2 summarizes the principal molecular and cellular mechanisms through which GLP-1RAs may influence periodontal health, with emphasis on inflammatory modulation, redox balance, bone metabolism, and regenerative pathways.

**Table 2:** Molecular and cellular actions of GLP-1 receptor agonists and their periodontal implications. GLP-1RA, glucagon-like peptide-1 receptor agonist; NF-κB, nuclear factor kappa-B; TNF-α, tumor necrosis factor-alpha; IL-6/IL-1β/IL-10, interleukins; AMPK, AMP-activated protein kinase; ROS, reactive oxygen species; ADMA, asymmetric dimethylarginine; AGE-RAGE, advanced glycation end-products/receptor for AGE axis; HDAC6, histone deacetylase 6; ERK1/2, extracellular signal-regulated kinases 1 and 2; Runx2, runt-related transcription factor 2; PKA, protein kinase A; β-cat, β-catenin; SOST, sclerostin; RANKL, receptor activator of NF-κB ligand; OPG, osteoprotegerin; TRAP, tartrate-resistant acid phosphatase; DPD, deoxypyridinoline; CTX, C-terminal telopeptide of type I collagen; PDLSC, periodontal ligament stem cell; P. gingivalis LPS, lipopolysaccharide of Porphyromonas gingivalis; ALP, alkaline phosphatase; MMP, matrix metalloproteinase; GCF, gingival crevicular fluid; T1D, type 1 diabetes; CAL, clinical attachment level; PD, probing depth; BOP, bleeding on probing Evidence tier: Preclinical = in vitro or animal studies; Human tissue/biomarker = human samples showing mechanistic modulation; Human metabolic/biomarker = systemic changes with periodontal implications inferred; Observational human = cohort/biomarker correlation data; RCTs pending = no completed periodontal randomized controlled trials yet.

Mechanism	Molecular/Cellular action	Key evidence/Model	Periodontal implication	Expected periodontal endpoints	Evidence tier
Anti-inflammatory (NF-κB, macrophages, cytokines)	GLP‑1RAs suppress NF‑κB activation and shift macrophage polarisation from M1 to M2; decrease TNF‑α, IL‑6, IL‑1β; increase IL‑10 [74-79,83].	Immune cells (macrophages/monocytes/lymphocytes) express GLP‑1R; chronic periodontitis shows upregulated NF‑κB; liraglutide reduces inflammatory infiltration and bone loss in ligature models [75,78,79,84].	Reduced gingival inflammation and pocket activity via attenuated pro‑inflammatory signaling [78,79,84-86]	BOP ↓, PD ↓	Preclinical; human tissue biomarker (NF‑κB upregulation)
AMPK activation / metabolic–immune crosstalk	GLP‑1RAs activate AMPK, attenuating inflammatory signaling and chemokine secretion; AMPK links metabolic control to inflammation resolution [74,80–82].	AMPK activation mitigates M1 polarization and cytokine release; periodontitis worsens in AMPK‑knockout mice [81,82].	Lower inflammatory tone in periodontal tissues through AMPK‑mediated pathways [80-82]	BOP ↓, PD ↓	Preclinical
Antioxidant (ROS, ADMA, autophagy)	GLP‑1RAs reduce ROS generation and ADMA via the AGE-RAGE axis; promote HDAC6 through GLP‑1R–ERK1/2 to curb ROS‑induced abnormal autophagy and inflammation [90–92].	ADMA is elevated in diabetes and periodontitis; higher GCF ADMA correlates with IL‑1β and clinical parameters [91,93,94].	Mitigates oxidative burden, supporting preservation of soft tissue and bone; expected reductions in BOP/PD and protection against alveolar bone loss [90-92,94]	BOP ↓, PD ↓, bone loss ↓	Preclinical; human biomarker (ADMA)
Osteogenesis (Wnt/β‑catenin, Runx2, ERK/Akt)	GLP‑1R signaling supports osteogenic differentiation via Wnt/β‑catenin, Runx2, ERK/Akt, and PKA/β‑catenin; suppresses sclerostin (SOST) [73,100–110].	Exendin‑4 restores Wnt signaling and bone formation in diabetic rats; MC3T3‑E1 cells show ERK1/2, PI3K/Akt, cAMP/PKA/β‑cat Ser675 activation with liraglutide [73,103].	Enhanced matrix maintenance and osteogenesis; potential CAL gains and crestal bone preservation [73,100–110]	CAL ↑, crestal bone preserved	Preclinical
Anti‑resorptive (RANKL/OPG balance)	GLP‑1RAs lower RANKL/OPG ratio, suppressing osteoclastogenesis [85,86].	Exendin‑4 upregulates OPG and suppresses RANKL mRNA in ovariectomized rats; ligature and diabetic models show bone loss reduction [72,84].	Fewer TRAP+ osteoclasts and reduced resorptive lacunae; stabilization of alveolar bone height [72,84-86]	Bone resorption ↓; bone height stabilized	Preclinical
Calcitonin‑mediated anti‑resorption	GLP‑1 enhances calcitonin release via the thyroid C‑cell GLP‑1R/cAMP pathway [113,114].	WT mice: exendin‑4 ↑, calcitonin; GLP‑1R‑deficient mice show ↓ calcitonin and ↑ urinary DPD; exendin‑4 reduces LPS‑induced osteoclast maturation/CTX [115,116].	Additional suppression of bone resorption, complementing local periodontal effects, particularly under inflammatory stimuli [115,116]	Bone resorption ↓	Preclinical
PDLSC‑mediated regeneration	Liraglutide promotes PDLSC migration/proliferation and osteogenic differentiation; counters Porphyromonas gingivalis LPS‑induced Wnt/β‑catenin perturbation; exendin‑4 protects PDLSC osteogenesis under high glucose [87,88,117-119].	In vitro PDLSC models (LPS challenge; high‑glucose) show ↑ ALP and Runx2 with reduced inflammatory cytokines [87,88,119].	Regenerative‑leaning environment that supports CAL improvement and alveolar bone stability [87,88,119]	CAL ↑, bone stability ↑	Preclinical (in vitro)
Anti‑MMP activity	GLP‑1RAs (exenatide, liraglutide, semaglutide) downregulate MMPs (incl. MMP‑9); GLP‑1 reduces IL‑1β‑induced MMP‑3 and MMP‑13 expression [89,122-125].	Type 1 diabetes: hyper/hypoglycemia spikes increase MMP‑9; GLP‑1 counterbalances these oxidative‑stress‑driven surges [126].	Preserves extracellular matrix and connective‑tissue attachment; may blunt episodic collagenolysis during exacerbations [126,127-133]	CAL loss ↓; collagen preservation ↑	Preclinical; human metabolic (MMP‑9 dynamics in T1D)
Biomarkers and monitoring	GCF commonly contains MMP‑8, MMP‑9, and MMP‑13; MMP‑8 accounts for ~80% of collagenase activity in GCF [127-131].	GCF MMP‑9 and MMP‑13 predict short‑term disease progression; osteoclasts express high MMP‑9 [134-137].	Non‑invasive markers to track host‑modulation (e.g., GLP‑1RAs) alongside BOP, PD, CAL, and radiographic change [137,135]	GCF MMP‑8/‑9/‑13 ↓ (target)	Human observational/biomarker
Preclinical periodontal outcomes	GLP‑1RAs reduce inflammatory‑gene induction and osteoclast development; they do not necessarily upregulate osteoblast activity in all models [84].	Ligature‑induced periodontitis and diabetic models: liraglutide decreases bone loss, improves microarchitecture, and lowers IL‑6, TNF‑α, and IL‑1β [84,121].	Likely improvements in PD, CAL, and crestal bone height in clinical cohorts; human RCTs with periodontal endpoints are still needed [121]	PD ↓, CAL ↑, bone height ↑ (expected)	Preclinical

Discussion

Inflammation is a shared feature of both diabetes and periodontitis. Diabetes influences periodontitis through four main mechanisms: it increases the immune-inflammatory response, impairs bone metabolism, induces oxidative damage, and promotes AGE-RAGE accumulation [140,141]. This four-pathway framework aligns with NF-κB/AMPK dysregulation and AGE-RAGE oxidative stress described in periodontal disease [79,80,91,140].

While GLP-1 levels are similar in both obese and non-obese patients with periodontitis, the levels increase following periodontal therapy. This suggests that oral inflammation has a greater impact on incretin hormone levels than obesity [141]. The rise in GLP-1 after treatment, independent of obesity status, indicates that inflammation acts as a key regulator of incretin modulation [141]. In another study, obese non-diabetic individuals with periodontitis displayed an impaired incretin axis with relative hyperglucagonemia, leading to worsened glucose tolerance and higher diabetes risk. GLP-1 levels were decreased in accordance with disease severity, while glucagon levels were increased. Alterations in oral and gut microbiota were hypothesized to accelerate dysglycemia and contribute to this imbalance. These findings position the incretin axis as a novel therapeutic target for preventing type 2 diabetes in patients with periodontitis [7]. Thus, targeting the incretin axis with GLP-1RAs may have preventive value against dysglycemia in this population [7]. It is important to note that GLP-1 exists in both basal and stimulated states. Under normal physiological conditions, circulating GLP-1 remains at low baseline levels (around 5-15 pmol/L) and rises postprandially to 20-50 pmol/L in response to nutrient intake [16,17]. In contrast, pharmacologic GLP-1RA therapy produces sustained elevations that far exceed these physiologic levels [18-22]. This distinction between normal and elevated states is essential for interpreting their systemic and periodontal implications. Importantly, there were improvements in incretin hormone levels after periodontal treatment, and this occurred without changes in weight, lipid profile, or blood pressure. Therefore, a direct link between oral inflammation and incretin is evident [141].

There is limited evidence of GLP-1R expression in gingival fibroblasts and periodontal ligament (PDL) cells. However, GLP-1R is well documented in immune cells such as macrophages, monocytes, and lymphocytes, which infiltrate inflamed periodontal tissues. This suggests that GLP-1RAs modulate local immune responses within the periodontium [74,76,80]. Animal studies, such as those demonstrating liraglutide-mediated reductions in M1 macrophage accumulation and gingival inflammation, further support the functional relevance of GLP-1R signaling in periodontal tissues [84]. The primary targets of GLP-1RAs may therefore be immune-lineage cells rather than fibroblasts or PDL cells, contributing to reductions in BOP and PD via immunomodulation [74,84].

Definitive GLP-1R mapping in human gingiva and PDL (e.g., via immunohistochemistry and single-cell RNA sequencing) is still lacking. Nonetheless, GLP-1RAs exert anti-inflammatory effects in periodontal inflammation through NF-κB inhibition and AMPK activation [74], both of which are strongly implicated in periodontal pathophysiology [79,81]. This dual modulation supports GLP-1RAs as a potential adjunctive anti-inflammatory therapy in periodontitis by suppressing cytokines such as IL-6, IL-1β, and TNF-α [77,81]. That said, the efficacy of GLP-1RAs may differ depending on their tissue penetration and immune-cell engagement.

Mechanistically, these actions are expected to improve clinical outcomes, including reductions in BOP, PD, and CAL, and reduced bone loss via RANKL/OPG regulation [72,79,81,121]. GLP-1 and GLP-1RAs also support bone metabolism, primarily through Wnt pathway activation, directing MSC differentiation toward osteoblasts, and upregulating Runx2 expression [100,103]. Animal studies have demonstrated that GLP-1RAs regulate the RANKL/OPG ratio by increasing OPG and reducing RANKL expression [72,121], thereby suppressing osteoclastogenesis. They also inhibit LPS-induced osteoclast maturation and bone resorption [116]. Collectively, these mechanisms may contribute toward attachment gain and crestal bone preservation [72,100,103,116,121].

Clinical evidence further supports this rationale. In a retrospective cohort of T2DM patients, those taking GLP-1 drugs had less peri-implant marginal bone loss at one year compared with insulin and metformin users, though no statistically significant difference remained at two years [142]. While observational, this suggests a potential peri-implant benefit from GLP-1Ras and warrants randomized trials with peri-implant bone endpoints [142]. In animal studies involving T2DM rats, exenatide enhanced peri-implant osseointegration [143]. Local delivery of exendin-4-loaded chitosan-PLGA microspheres promoted peri-implant bone formation and osseointegration without affecting blood glucose levels [144], consistent with a direct peri-implant tissue effect.

In studies done in vitro, exenatide and liraglutide facilitated the osteogenic differentiation of PDLSCs via Wnt and MAPK pathways, even under hyperglycemic or inflammatory conditions [87,119]. These findings reinforce the dual local and systemic mechanisms by which GLP-1RAs may benefit periodontal and peri-implant health.

While the mechanistic and translational evidence is encouraging, there is still a lack of randomized human trials that investigate periodontal endpoints, such as BOP, PD, CAL, and radiographic bone changes. Accordingly, the periodontal benefits of GLP-1RAs should be regarded as promising but unproven, as they are supported primarily by preclinical and indirect metabolic evidence. Clearly defining these endpoints in future trials will be essential to distinguish glycemia-independent effects from those driven purely by metabolic improvements.

In summary, studies in animal models demonstrate that GLP-1RAs alleviate periodontal inflammation, reduce oxidative stress, and decrease alveolar bone resorption, while enhancing bone remodeling and implant osseointegration. These benefits are evident even in the absence of glycemic changes [72,84,103,115,116,121,143,144]. Human studies should be conducted to translate these findings into clinical practice. Periodontal endpoints (BOP, PD, CAL, and radiographic bone changes) should be specified and GCF biomarkers (MMP-8/9/13) incorporated. To confirm glycemia-independent effects, studies should adjust for confounders such as HbA1c and weight [134,135,142,144]. Table 3 integrates experimental, observational, and translational findings on the interplay between GLP-1RAs, diabetes, and periodontal/peri-implant health, highlighting mechanisms, implications, and research priorities.

**Table 3:** Evidence linking GLP-1/GLP-1RAs to periodontal and peri-implant health outcomes. GLP-1RA, glucagon-like peptide-1 receptor agonist; GLP-1R, glucagon-like peptide-1 receptor; NF-κB, nuclear factor kappa-B; AMPK, AMP-activated protein kinase; IL-6/IL-1β/TNF-α, interleukins/tumor necrosis factor-alpha; AGE–RAGE, advanced glycation end-products/receptor for AGE axis; GCF, gingival crevicular fluid; PPG, post-prandial glucose; Runx2, runt-related transcription factor 2; Wnt/β-catenin, canonical bone-formation signalling pathway; RANKL/OPG, receptor activator of NF-κB ligand/osteoprotegerin; PDLSC, periodontal ligament stem cell; LPS, lipopolysaccharide; T2DM, type 2 diabetes mellitus; ALP, alkaline phosphatase; ISQ, implant stability quotient; IHC, immunohistochemistry; scRNA-seq, single-cell RNA sequencing; MMP, matrix metalloproteinase; CAL, clinical attachment level; PD, probing depth; BOP, bleeding on probing Evidence tier: Preclinical = animal or in vitro models; Human observational/metabolic = clinical cohorts or biomarker/metabolic correlation studies; Human tissue biomarker = mechanistic findings in human periodontal tissue; Evidence gap/research priority = areas requiring dedicated periodontal RCTs and molecular mapping. Endpoints and biomarkers: periodontal clinical measures (BOP, PD, CAL, marginal bone loss), radiographic bone preservation, osseointegration indices (ISQ, bone-to-implant contact), and biochemical markers (cytokines, RANKL/OPG, ALP, MMP-8/-9/-13).

Mechanism/pathway	Key findings (with citations)	Periodontal/Peri-implant implications	Evidence tier	Endpoints and biomarkers to track	Confounders to adjust
Inflammation and immune modulation	Diabetes worsens periodontitis via ↑ inflammatory response, impaired bone metabolism, oxidative stress, and AGE–RAGE signaling [79,80,91,141]; GLP‑1RAs inhibit NF‑κB and activate AMPK, suppressing IL‑6, IL‑1β, and TNF‑α [74,77,81].	Reduced gingival inflammation; expected improvements in BOP, PD, CAL via attenuated pro‑inflammatory signaling [72,79,81,121]	Preclinical; human tissue biomarker (NF‑κB upregulation in periodontitis)	BOP ↓, PD ↓, CAL ↑; optional: GCF cytokines (IL‑1β/IL‑6/TNF‑α)	HbA1c, weight, smoking
Incretin axis dysregulation	GLP‑1 levels rise after periodontal treatment independent of obesity, indicating an oral‑inflammation → incretin link [142]; obese periodontitis patients show impaired incretin axis with hyperglucagonemia and low GLP‑1, worsening glucose tolerance [7].	GLP‑1RAs may help prevent dysglycemia in periodontitis, highlighting the incretin axis as a therapeutic target [7,142]	Human observational/metabolic	BOP, PD, CAL; GLP‑1 and glucagon levels; glycemic metrics (fasting/PPG/HbA1c)	Weight change, baseline glycemia, meds (metformin/insulin), microbiome factors
Bone remodeling and osteogenesis	GLP‑1RAs promote bone metabolism via Wnt/β‑catenin and Runx2 activation [100,103]; regulate RANKL/OPG balance [72,121]; inhibit LPS‑induced osteoclastogenesis [116]; enhance PDLSC osteogenesis under hyperglycemia/inflammation [87,119].	Supports bone remodeling, attachment gain, and crestal bone preservation [72,100,103,116,121]	Preclinical (in vivo and in vitro)	CAL ↑, radiographic crestal bone preserved; biomarkers: RANKL/OPG, ALP, Runx2	HbA1c, vitamin D/calcium status, smoking
Peri‑implant osseointegration	GLP‑1 users had less peri‑implant bone loss vs insulin/metformin at 1 year [142]; exenatide enhances osseointegration in T2DM rats [143]; local exendin‑4 microspheres improve bone formation independent of glycemia [144].	Potential improvement in peri‑implant bone healing and osseointegration [142-144]	Human observational; animal; local‑delivery preclinical	Marginal bone loss (MBL), ISQ/resonance frequency, bone‑to‑implant contact	HbA1c, implant site quality, periodontitis history, smoking
Translational gap	GLP‑1R confirmed in immune cells within periodontal lesions, but mapping in the gingiva/PDL remains incomplete [74,76,80].	Need definitive human studies with periodontal endpoints and molecular mapping (IHC/scRNA‑seq) [134,135,142,144]	Evidence gap/research priority	BOP, PD, CAL, radiographic bone change; GCF MMP‑8/‑9/‑13; GLP‑1R localization; NF‑κB/AMPK readouts	HbA1c, weight, medications, smoking, baseline disease stage

Future research directions of GLP-1RAs in periodontal health

Given their established efficacy and safety in managing obesity and diabetes, GLP-1RAs may also hold therapeutic potential for periodontitis. This dual approach could improve systemic metabolic parameters (HbA1c reduction, weight loss) while supporting periodontal health through reduced inflammation and bone preservation. However, most available data are derived from in vitro or animal studies.

This review has analyzed diverse preclinical and limited human evidence, but study comparability is limited by heterogeneity in GLP-1RA class, dosing, co-therapies, and periodontal case definitions. Publication bias and selective reporting are also possibilities. Furthermore, most mechanistic pathways (NF-κB/AMPK, RANKL/OPG, Wnt/β-catenin) have only been theorized to have periodontal endpoints; confirmation is required via randomized human trials.

Future research should prioritize well-designed human clinical trials that prespecify periodontal endpoints such as BOP, PD, CAL, and radiographic bone changes. Biomarkers including GCF MMP-8/9/13 and RANKL/OPG should be integrated, and HbA1c and weight should be adjusted for to isolate direct tissue effects [55-56,66-69,72,121,134,135,142].

In addition to systemic use, local application of GLP-1RAs as adjuncts to non-surgical periodontal therapy should be investigated. Prior studies in T2DM rats demonstrated that exendin-4-loaded chitosan-PLGA microspheres enhanced peri-implant osseointegration. This was done without affecting glycemia, thus supporting the feasibility of local adjunctive delivery [144].

Although GLP-1RAs display promising anti-inflammatory, osteoprotective, antioxidant, and anti-MMP properties, direct evidence of their roles in oxidative stress control and MMP regulation in periodontitis remains limited. Future molecular studies should map GLP-1R expression in gingiva and PDL (e.g., immunohistochemistry, single-cell RNA sequencing) and integrate transcriptomic/proteomic readouts to link NF-κB/AMPK and ROS/MMP modulation to clinical outcomes [74,80,90-92,127-135].

Ultimately, the development of GLP-1RAs, whether locally delivered or used systemically as an adjunctive, should focus on patients with metabolic syndrome and periodontitis. This will pave the way toward a personalized medicine approach for the diabetes-obesity-periodontitis triad. Their potential as monotherapy for periodontitis should also be considered, taking into account possible side effects. In addition, future trials should stratify participants by obesity or diabetes status and periodontitis stage. They can then employ mediation analyses to distinguish glycemia and weight effects from local periodontal actions, consistent with observed differential incretin patterns in periodontitis cohorts [7,141].

Clinical implications for dental practice

For patients receiving GLP-1RAs, dental practitioners should adopt a comprehensive approach that integrates systemic and local considerations. A full record of the agent prescribed, including its dose, timing, and any recent adjustments, is essential. Practitioners should be aware of gastrointestinal side effects such as nausea, early satiety, and delayed gastric emptying, as these may influence both erosion risk and the provision of oral-hygiene advice. Xerostomia should be managed proactively with hydration strategies, sugar-free gum or xylitol, high-fluoride toothpaste or varnish, and maintenance intervals of three to four months. Reinforcement of home-care practices is particularly important during dose-escalation phases when side effects may be more pronounced. In addition, clinicians should remain mindful of peri-implant implications, since preclinical and observational evidence suggests that GLP-1RA therapy may support favorable bone remodeling. For high-risk patients, monitoring inflammatory and biomarker responses can complement standard periodontal assessments as part of comprehensive care. These recommendations align with the systemic effects of GLP-1RAs on glycemic control, weight, and appetite, as well as their local influence on immuno-bone pathways. Importantly, they are intended as supportive measures and not substitutes for conventional periodontal therapy [55-69,72,74-81,84,87-90,103,115,116,121,122-135].

## Conclusions

GLP-1RAs are widely used in the management of diabetes and obesity, but they demonstrate potential benefits that extend beyond glycemic control. Preclinical and experimental evidence highlights their anti-inflammatory, antioxidant, osteoprotective, and anti-MMP properties, all of which are relevant to the preservation of periodontal and peri-implant health. While GLP-1RAs do not directly restore periodontal homeostasis, they appear to help maintain periodontal tissue integrity in individuals with metabolic comorbidities such as diabetes and obesity.

Findings from animal and in vitro studies suggest that GLP-1RAs may serve as either systemic or localized adjuncts in periodontal therapy. However, robust human clinical trials are needed to confirm their clinical effectiveness and to establish therapeutic strategies for managing the interrelated conditions of periodontitis, diabetes, and obesity. Beyond the extensively studied liraglutide and exenatide, future research should also evaluate the periodontal effects of newer GLP-1RAs to broaden the evidence base and guide clinical use.
